# Differences in the Composition of Vaginal Microbiota between Women Exhibiting Spleen-Deficiency Syndrome and Women with Damp-Heat Syndrome, Two of the Most Common Syndromes of Vaginitis in Traditional Chinese Medicine

**DOI:** 10.1155/2019/5456379

**Published:** 2019-10-24

**Authors:** Wei Xian Lin, Xin Du, Li Lin Yang, Si Yun Chen, Wei Yu Qiu, Hai Wang Wu, Guang Zhao, Yi Hui Feng, Qing Ying Yu, He Tian, Song ping Luo, Jie Gao

**Affiliations:** ^1^First School of Clinical Medicine, Guangzhou University of Chinese Medicine, Guangzhou, China; ^2^Tongji Medical College, Huazhong University of Science and Technology, Wuhan, China; ^3^Department of Obstetrics and Gynecology, First Affiliated Hospital of Guangzhou University of Chinese Medicine, Guangzhou, China

## Abstract

Spleen-deficiency syndrome and damp-heat syndrome are the two most common syndromes of vaginitis in traditional Chinese medicine (TCM). Although it is known that the vaginal microbiota is closely associated with vaginitis, present studies have not fully elucidated the relationship between the composition of the vaginal microbiome and type of TCM syndrome because of the limitations in the present reductionist approaches. Samples of vaginal secretions were collected from patients with bacterial vaginitis and healthy subjects with spleen-deficiency syndrome and damp-heat syndrome, in order to analyze the constitution of the vaginal microflora using 16S rRNA sequencing methods that encompass taxonomic units, alpha diversity rarefaction curves, and principal component analyses. This prospective study indicated that there was a statistically significant difference in the composition of the vaginal microbiome between patients with spleen-deficiency syndrome and patients with damp-heat syndrome. *Streptococcus* was the dominant microbiota in patients with spleen-deficiency syndrome. This can serve as a biomarker for differentiating spleen-deficiency syndrome and damp-heat syndrome. In addition, as indicated by the findings on the samples, patients with bacterial vaginitis of dominant abundance in *Pseudomonadaceae* might be prone to manifest spleen-deficiency syndrome, while patients with bacterial vaginitis of dominant abundance in *Prevotella* might be prone to manifest damp-heat syndrome. These present findings can provide a new approach to acquire a scientific understanding of the syndromes of TCM, which in turn would benefit the development of personalized medicine, in terms of ancient medicine and complex biological systems.

## 1. Introduction

Vaginitis is one of the most common gynecological diseases, and approximately 5%–70% of women suffer from bacterial vaginitis (BV) [1]. The most common dysbiosis of the vaginal microbiome is BV, and the symptoms of BV mainly include vaginal itching and the production of a whitish-grey discharge with an unpleasant odor, which causes great inconvenience to a patient's life [2, 3]. Women with vaginal symptoms and diagnosed with BV would empirically receive antibiotic treatment, which could destroy the ecosystem in the vagina. In addition, due to the extensive use of antifungal agents and antibiotics, drug resistance has become a problem that awaits a solution. Hence, there is a pressing need to find new treatments.

Traditional Chinese medicine (TCM) has a history of over 3,000 years. Over centuries of empirical practice, practitioners of TCM have found that treatments for vaginitis based on the TCM theory could yield a satisfactory therapeutic effect. Clinical research has revealed that the treatment of vaginitis using vaginal lavage with TCM has demonstrated satisfactory efficacy [4, 5]. In the TCM theory, vaginitis is usually classified as the area of “leukorrheal diseases,” and the treatment is mainly based on syndrome differentiation. Syndrome (“Zheng” in Mandarin Chinese) is a key principle in TCM. Based on the TCM theory of syndrome differentiation, TCM practitioners typically classify patients afflicted with the same disease into subgroups of different syndromes from a holistic perspective on the overall status of patients. According to the theory of TCM, the causative agent/factor of vaginitis can be summarized as heat, damp, cold, and toxin. In addition, the salient feature of TCM is holism, with emphasis in regulating the integrity of the human body, as well as the interaction between human individuals and the environment. In Guangzhou, China, owing to the extremely hot-damp weather, “damp” has become the major factor in vaginitis, which leads to pathological changes. Thus, spleen-deficiency syndrome and damp-heat syndrome have become the most common syndromes observed in vaginitis clinics. According to the TCM theory, the treatment is based on the holistic concept and the theory of syndrome differentiation. At present, owing to the clinical advances in anti-inflammatory and antibacterial, TCM has made dramatic progress and contributions in the understanding and treatment of numerous diseases. With the use of systems biology as a new route, the study of TCM, in a holistic manner, has become one of the popular research topics in modern science.

Recently, with the application of high-throughput molecular methods, including next-generation sequencing (NGS), it was revealed from the microscopic examination data that the microbiome is more complex than previously thought and that the development of NGS technology for characterizing microbial communities provides an opportunity to further understand the impact of the microbiome. Some studies have indicated that the microbial community in the human body plays a role in human physiology and pathology [6, 7]. For instance, the tongue microbiome is implicated in gastritis, cancer, precancerous lesions and intra-oral halitosis [8–10]. Furthermore, several studies have used NGS to analyze vaginal microbiomes in various diseases, including idiopathic infertility and vaginitis [11, 12]. Although these studies have compared the NSG results of microbiomes in different diseases, no study has compared NGS results with TCM syndromes. The question that arises is whether a connection exists between the vaginal microbiome and TCM syndrome. It was hypothesized that the composition of the vaginal microbial community is associated with the TCM syndrome.

In the present study, vaginal secretions were collected from BV patients who manifested with spleen-deficiency syndrome and damp-heat syndrome, and from healthy volunteers who had vaginal symptoms and manifested with spleen-deficiency syndrome and damp-heat syndrome. The microbiomes in the vaginal secretion were sequenced using the 16S ribosomal RNA (16S rRNA) sequencing analysis. The present study furnished information for the practitioners to consider primary prevention based on the theory of preventive treatment of diseases in TCM. The present study provides a new approach for understanding the scientific basis of TCM syndromes and serves primarily to build a bridge between TCM and molecular systems biology.

## 2. Methods

### 2.1. The Included Samples

**Figure 1:**
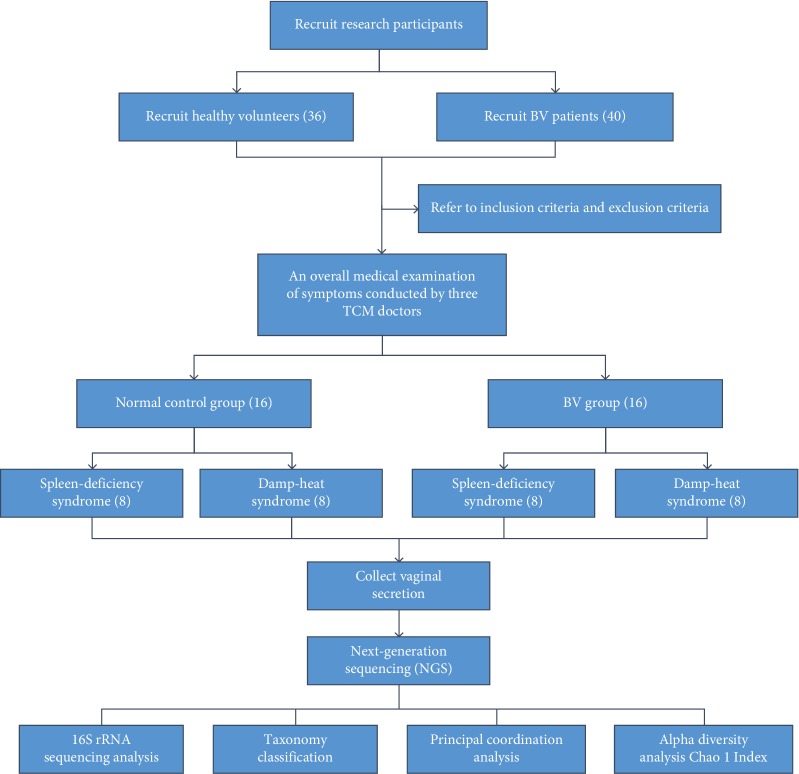
Study design flow chart.

**Figure 2:**
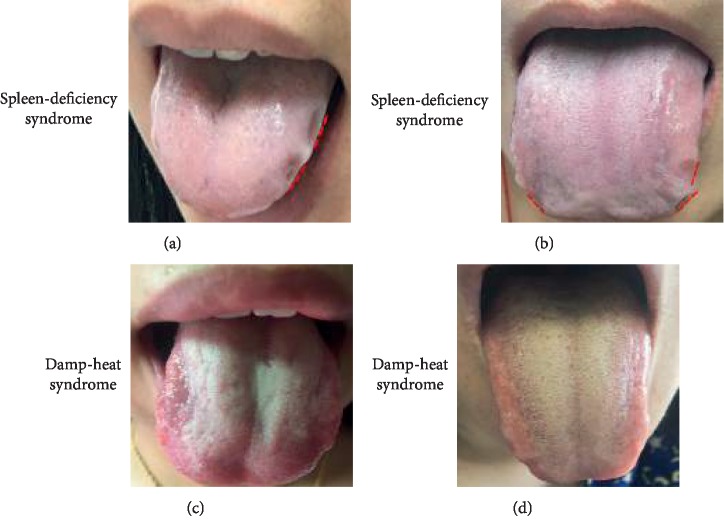
TCM tongue-coating appearance classification. (a, b) Typical tongue-coating of spleen-deficiency syndrome, pale tongue with teeth marks (red dotted) and white coating. (c, d) Typical tongue-coating of damp-heat syndrome, red tongue, and yellow greasy coating.

The present study was approved by the Medical Ethics Committee of the First Affiliated Hospital of Guangzhou University of Chinese Medicine. All patients and healthy volunteers provided informed consent. This was a prospective study; all participants in the present study had vaginal symptoms, were within 20–40 years old, and had a medical history. Participants who used glucocorticoids and antibiotics in the previous month, and subjects who had smoking habits or alcohol abuse in the past three months, and had sexual intercourse in the past three days were excluded. A total of 76 participants, which included 36 healthy volunteers and 40 BV patients, were recruited, and a total of 32 participants who met the criteria were included in the study. The diagnosis of BV was based on the guidelines of the Infection Disease Society of America [1]. According to the diagnosis, patients were divided into two groups: control group (*n* = 16) and BV group (*n* = 16). These two groups were further divided into two subgroups: spleen-deficiency syndrome and damp-heat syndrome.  Figure 1 presents the entire process of the present clinical study. The identification of Chinese medical syndromes was determined by two Deputy Directors, who are Chinese medicine practitioners, and based on the Gynecology of traditional Chinese medicine [13]. The tongue image is one of the most important diagnostic markers of TCM syndromes.  Figure 2 presents the typical tongue images of the spleen-deficiency syndrome (Figures 2(a) and 2(b)) and damp-heat syndrome (Figures 2(c) and 2(d)), respectively. The samples of vaginal secretion were collected by injecting 50 ml of normal saline to the vagina, waiting for one minute, and sucking 40 ml of fluid. The collected sample was stored in a centrifuge tube at −80°C before extraction.

### 2.2. The 16S rRNA

Total DNA was extracted using a Hipure Bacterial DNA kit (Magen, D3146; China), according to manufacturer's instructions. This was inspected using an ultramicrospectrophotometer (K5500, Kai'ao, China), and by agarose gel electrophoresis (Qubit® dsDNA HS Assay Kit, Life Technologies, USA; Qubit 2.0, Life Technologies, USA). Universal primers for 16S variable regions V3-V4 were used for the polymerase chain reaction (PCR) amplification. The specific primers inside and outside were amplified by multiplex PCR assay (NEBnext-Ultra-II-Q5-Master-Mix, NEB, USA) and inspected by employing the Agilent 2200 Tapestation system (Aglient Technologies, USA). These samples were prepared according to the Miseq User Guide (Miseq Reagent Kit V3; 600 Cycle PE, Illumina, USA), which mainly included four steps: library preparation, cluster generation, sequencing, and data analysis. Subsequently, the sequencing of the paired-end (2X300) was conducted according to the Miseq User Guide.

### 2.3. Data Preprocessing

Data preprocessing was carried out as follows: (i) Screening of low-quality data. (ii) The remaining paired-end reads were assembled into unique tags by FLASH (Version 1.2.6), the overlapping of the forward and reverse ends was performed to obtain the 16S V3-V4 tags, and the chimera was wiped out. High-quality, nonchimeric V3-V4 tags were acquired from the raw paired-end reads. Similar sequences were searched from the Greengenes database using QIIME (Version 1.0), in order to obtain the operational taxonomic units (OTUs). Then, the species abundance and bacterial colonies were assessed based on the phylum, as annotated by the tags. In the alpha diversity analysis, the Chao1, Shannon Index, and Simson index were calculated. Alpha diversity was generally used to predict the amount of microorganisms by estimating the index of OTUs. In the beta diversity analysis, the distance matrix was calculated using the Bray–Curtis formula. The unweighted unifrac was also analyzed. Principal Coordinate Analysis (PCoA), a matrix calculated by beta variance analysis, was used to visualize the different major components. In addition, clustering analysis was carried out using the weighted and unweighted pair-group method with arithmetic means. In order to determine whether the clustering patterns observed in the nonmetric multidimensional scaling (NMDS) plots were statistically supported by the differences in the distance matrix, an analysis of molecular variance (AMOVA) [14] implemented by mothur was performed. The significant difference among groups was calculated with the aid of the Metastats software. After normalizing the data and using *t*-test, *P*-values were obtained.

## 3. Results

### 3.1. Patient Enrollment and Sample Collection

All participants had vaginal symptoms, and a total of 32 samples of vaginal secretion were collected. According to Western medicine, these patients were divided into two groups: BV group, patients diagnosed with BV (*n* = 16); control group, healthy control women (*n* = 16). In the viewpoint of TCM, these patients were categorized into two subtypes, based on the overall medical examination of symptoms conducted by two TCM doctors: spleen-deficiency syndrome (*n* = 16) and damp-heat syndrome (*n* = 16) (Table 1). Despite the common clinical manifestations of these participants, such as vaginal itching and abnormal leucorrhea, patients with spleen-deficiency syndrome and damp-heat syndrome exhibited characteristic differences. Tongue-coating appearance is one of the most significant features employed in the differentiation of syndromes. The representative tongue-coating images obtained from two different syndromes are presented in Figure 2. For the selection of samples in the present pilot study, patients without vaginal lavage or patients who did not receive medicine within three months prior to the examination, and patients who used condoms during intercourse were chosen. Meanwhile, the age of these patients was well-matched (approximately 28 years old and 29 years old, respectively) for both diseases, as diagnosed by Western medicine and the syndromes classified by TCM, which are spleen-deficiency syndrome and damp-heat syndrome (Table 2).

### 3.2. The 16S rRNA Sequencing Analysis and Taxonomy of the Vaginal Microbiome

**Figure 3:**
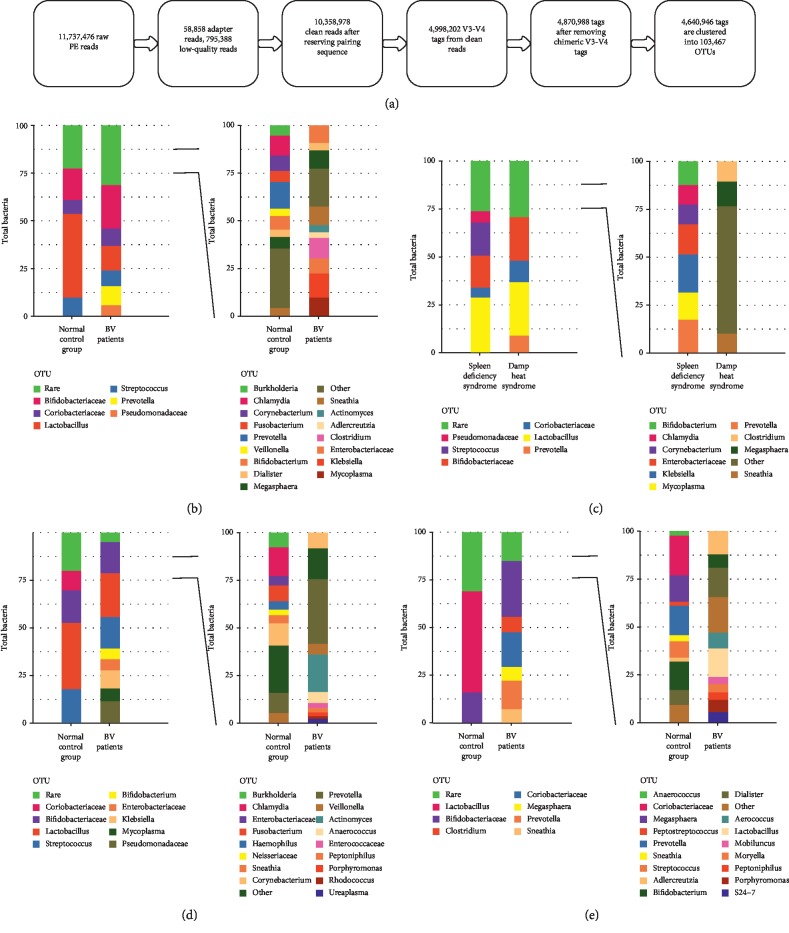
16S rRNA sequencing analysis and taxonomy classification of the vaginal microbiome at the genus level. (a) The flow chart of sequence preprocessing, quality control and chimera detection. (b) Relative abundance of dominant and rare genus in samples of healthy volunteers and BV patients. (c) Relative abundance of dominant and rare genus in samples with different TCM syndromes. (d) Relative abundance of dominant and rare genus in spleen-deficiency syndrome of healthy volunteers and BV samples. (e) Relative abundance of dominant and rare genus in damp-heat syndrome of healthy volunteers and BV samples. PE: paired-end, OTU: operational taxonomic units, BV: bacterial vaginitis.

After filtering the reads and reserving the pairing sequences of 32 samples, 10,358,978 clean reads were spliced into 4,998,202 V3-V4 tags. Then, 103,467 OTUs were clustered for downstream analysis after removing the chimeric V3-V4 tags (Figure 3(a)). Given the tremendous importance of the functional roles played by bacteria, the vaginal microbiome was examined at the genus level. Among the classification of bacterial groups revealed by these interpretable sequences, the vaginal microbiomes of healthy volunteers and patients with BV were dominated by a total of four genera (relative abundance ≥5%): *Lactobacillus* (relative abundance: 43.88% in healthy volunteers and 12.83% in BV patients), *Bifidobacteriaceae* (relative abundance: 16.54% in healthy volunteers and 22.81% in BV patients), *Streptococcus* (relative abundance: 9.82% in healthy volunteers and 8.26% in BV patients), and *Coriobacteriaceae* (relative abundance: 7.22% in healthy volunteers and 9.14% in BV patients). Meanwhile, the dominated abundance of *Prevotella* (9.95%) and *Pseudomonadaceae* (5.82%) was observed in BV patients (Figure 3(b)). There were three main genera in both spleen-deficiency syndrome and damp-heat syndrome: *Lactobacillus* (28.87% for spleen-deficiency syndrome and 27.84% for damp-heat syndrome), *Bifidobacteriaceae* (16.70% for spleen-deficiency syndrome and 22.66% for damp-heat syndrome), and *Coriobacteriaceae* (5.14% for spleen-deficiency syndrome and 11.22% for damp-heat syndrome). In addition, *Pseudomonades* (5.89%) and *Streptococcus* (17.21%) were the abundant genera in subjects with spleen-deficiency syndrome, while *Prevotella* (9.05%) was the abundant genera in subjects with damp-heat syndrome (Figure 3(c)). The three common vaginal genera, which were *Lactobacillus* (34.78% in healthy volunteers and 22.96% in BV patients), *Streptococcus* (17.91% in healthy volunteers and 16.52% in BV patients), and *Bifidobacteriaceae* (17.05% in healthy volunteers and 16.34% in BV patients), were all abundant in the control and BV groups with spleen-deficiency syndrome. In addition, the dominant vaginal microbiomes in BV patients with spleen-deficiency syndrome at the genus level were *Pseudomonadaceae, Bifidobacterium*, *Enterobacteriaceae*, *Klebsiella*, and *Mycoplasma* (Figure 3(d)). Meanwhile, by examining the microbiomes in the two groups of damp-heat syndrome, it was observed that *Bifidobacteriaceae* (16.03% in healthy volunteers and 29.28% in BV patients) was abundant in healthy volunteers and BV patients, while *Prevotella* was specificity expressed in BV patients (Figure 3(e)).

### 3.3. Vaginal Microbiome in Spleen-Deficiency Syndrome and Damp-Heat Syndrome

**Figure 4:**
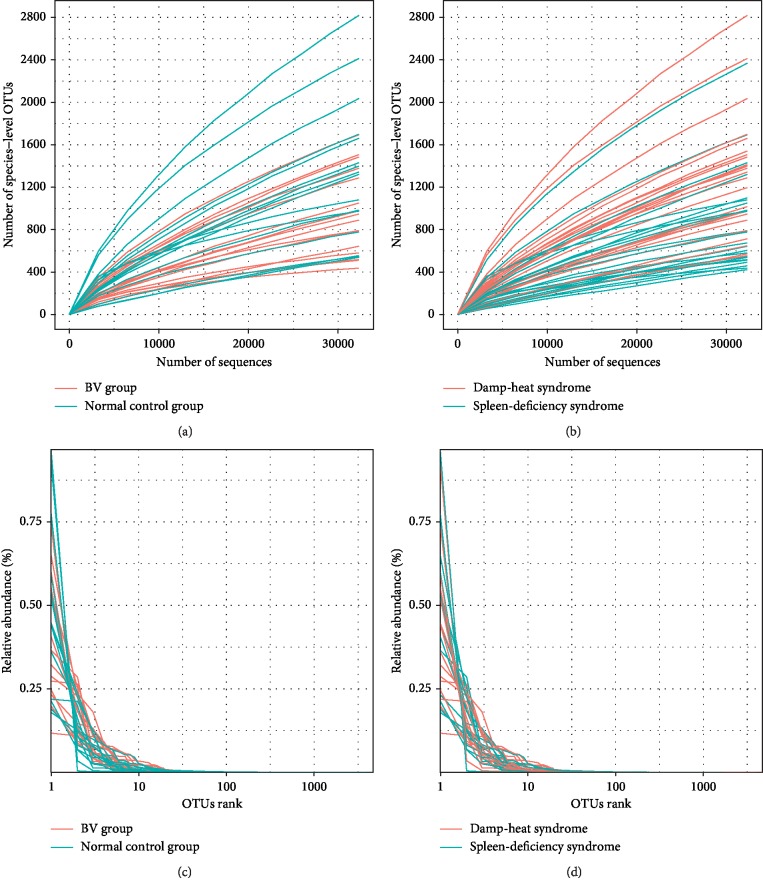
Alpha diversity rarefaction curves of samples based on species-level OTUs. (a) The rarefaction curves of different groups. (b) The rarefaction curves of different syndromes. (c) The rank abundance curves of different groups. (d) The rank abundance curves of different syndromes.

**Figure 5:**
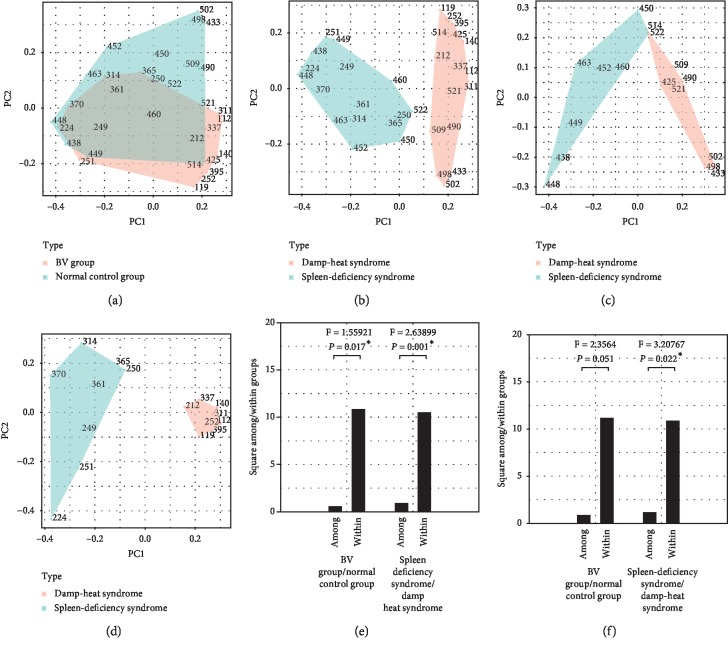
PCoA of the dissimiliarities among bacterial community taxonomical structure using Unifrac distance. (a) PCoA plot of the samples among healthy and BV patients. (b) PCoA plot of the samples between spleen-deficiency syndrome and damp-heat syndrome. (c) PCoA plot of the healthy volunteers' samples between the two syndromes. (d) PCoA plot of the BV patients' samples between the two syndromes. (e) AMOVA results of the BV and normal control group by unweighted Unifrac distance. (f) AMOVA results of the BV and normal control group by weighted unifrac distance. PCoA: principal coordination analysis, AMOVA: analysis of molecular variance. (^*∗*^represents *P* ≤ 0.05, PC1 represents the first principal component, PC2 represents the second principal component).

**Figure 6:**
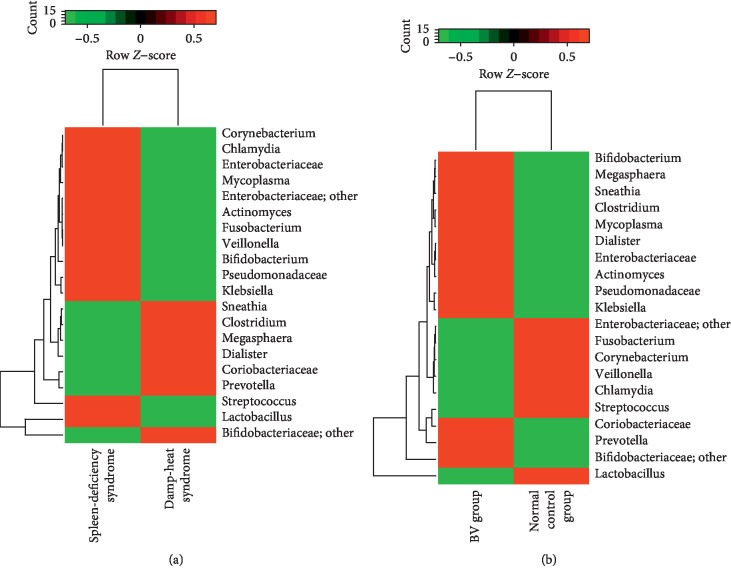
Heatmap representing the differences between the two syndromes and the two groups. (a) Heatmap of patients' samples with spleen-deficiency syndrome and damp-heat syndrome. (b) Heatmap of BV and normal control group.

Alpha diversity analyses were further conducted to identify the features of the vaginal microbiota composition. Every rarefaction curve was drawn at a specific OTU level (Figures 4(a) and 4(b)) as a function of the observed number of OTUs on sequence counts by resampling at different sequencing depths. The rank abundance curve is a way of representing diversity of vaginal microbiome (Figures 4(c) and 4(d)). In the sample, each OTU is sorted by abundance from large to small with the sort number of OTU as the abscissa, and the relative percentage of the number of sequences in the OTU is plotted on the ordinate. Furthermore, the PCoA method [14] was used to observe species with important contributions, in order to determine whether there are differences in the OTU compositions of vaginal microbiomes between healthy subjects and BV patients, and between the two syndromes. Through the Bary–Curtis formula and in calculating the Unifrac distance, the PCoA plots of the samples were completed (Figures 5(a)–5(d)). This demonstrated that the features of the vaginal microbiota were significantly different between patients with spleen-deficiency syndrome and patients with damp-heat syndrome. Furthermore, by using these two types of distance measures, it was discovered that there were significant differences, not only among the samples obtained from healthy subjects and BV patients (*P*=0.017, Figure 5(e)) but also between the two syndromes, within an unweighted Unifrac distance matrix (*P*=0.001, Figure 5(e)). Under the condition of weighted Unifrac distance, there was a highly statistically significant difference between syndromes (*P*=0.022, Figure 5(f)). Therefore, from the perspective of both species (unweighted Unifrac) and species and abundance (weighted Unifrac), there were statistically significant differences. Furthermore, in order to examine the clustering patterns of samples obtained from patients, a hierarchical clustering heatmap was generated based on all bacterial communities across all samples. These analyses revealed that samples obtained from patients with spleen deficiency syndrome were very different from samples obtained from patients with damp-heat syndrome at the genus level (abundance >20, Figure 6). Furthermore, the samples obtained from patients with damp-heat syndrome had a greater abundance of *Prevotella*, *Bifidobacteriaceae*, *Coriobaceriaceae*, *Dialister*, *Megasphaera*, *Clostridium*, and *Sneathia*, when compared with samples obtained from patients with spleen-deficiency syndrome.

## 4. Discussion

Tracing back to the long history of traditional clinical practice in China and other Eastern countries, the concept of holism, and treatment based on syndrome differentiation has been the core therapeutic principle. Benefited by the accumulation of clinical experience of thousands of years, the practice of TCM has improved through observation and testing and provoked unique critical thinking for treating diseases. Particularly, the TCM syndrome, which is also called zheng or pattern, is the key concept of TCM theory, and this is based on clinical information. TCM practitioners could typically classify patients affected by the same disease into subgroups with different syndromes (zheng) through four main TCM diagnostic procedures: observation, listening, questioning, and pulse analysis [15]. According to the TCM syndrome differentiation, patients suffering from the same disease might be classified into different syndromes, while different diseases might be categorized as the same syndrome. In addition, TCM syndrome differentiation is dynamic, because one patient at different points in time may present with different syndromes, and syndromes can change during the evolution of a disease. In summary, TCM syndrome differentiation can be considered as a disease diagnostic guideline to help clinicians in the further stratification of patients with one disease, and obtain more accurate patient classifications.

At present, the diagnosis of TCM syndromes is combined with biomedical diagnosis in clinical practice, and integrative medicine has emerged as an optimal approach to achieve higher efficacy [15]. An increasing number of studies have made considerable achievements in proving syndrome-based efficacy, and these have shown that some TCM syndromes tend to result in particular diseases. TCM syndrome differentiation has been used for developing treatments in clinical trials. For instance, TCM syndrome is similar to the “microscopic concept” of epigenetics, in which the biological basis of chronic hepatitis B syndrome differentiation from the perspective of epigenetics is of great significance to diagnose and prevent diseases [16]. It has also been demonstrated that in patients with acute leukemia, when compared with the dual vacuity of the Qi and Yin syndrome, ID4 gene methylation is more likely to occur in patients with toxic hot flaming syndrome and static blood and binding phlegm syndrome [17]. In addition, the existence of a TCM syndrome may affect tumor growth in pancreatic cancer *via* specific chemokines and their receptors [18]. A previous study also disclosed that the tongue-coating microbiome is a novel holistic biomarker for characterizing patients with different syndromes that reflect gastrointestinal diseases [6]. Furthermore, a number of studies have revealed that TCM syndromes differentiate biomedical diseases into different patterns and that each pattern comprises a symptom that has its unique treatment [15]. In conclusion, these studies all indicate that clinical practice with TCM syndrome differentiation can lead to the improvement and innovation of biomedical diagnoses.

The vaginal microbiota is correlated to physiological balance in mammals [19]. These prevent vaginal infections, such as aerobic vaginitis, bacterial vaginitis, and candidiasis [20, 21]. Furthermore, the composition of the vaginal microbiota varies with age: when women reach the age of menopause, an obvious decrease in the number of lactobacilli is observed [22–27]. Furthermore, it has been reported that the composition of the vaginal microbiota is correlated with the risk of preterm birth [28] and that an increased risk of preterm birth is associated with high levels of *Lactobacillus* spp. [29], as well as several anaerobic bacteria and various Bacteroidetes [30–32]. It has been reported that without inducing inflammation, lactobacilli may maximize successful pregnancy outcome [33]. However, there are very few reports on the relationship between the composition of the vaginal microbiota and TCM syndrome. Therefore, it was hypothesized that a relationship might exist between these two aforementioned syndromes in disease-free women with local symptoms and BV patients and that different syndromes may reflect the features of the vaginal microbiota, which are associated with the status of the human body. In order to test this hypothesis, an NGS analysis of vaginal microbiome samples obtained from 16 disease-free women and 16 patients with BV was carried out. These two groups, each comprised eight patients with spleen-deficiency syndrome and eight patients with damp-heat syndrome.

The results of the statistical analysis of these sequences demonstrate the potential relationship between the composition of the vaginal microbiota and traditional syndrome differentiation, and the possible connection between different syndromes, which involve spleen-deficiency, damp-heat syndrome, and the microbiota of BV patients. Furthermore, these results revealed that the abundance of *Streptococcus* determined whether spleen-deficiency syndrome or damp-heat syndrome has occurred. Simultaneously, the decreased abundance of *Lactobacillus* in the microbiota of BV patients was found in the present study, which is consistent with the results of previous reports [34–36]. We also found that *Lactobacillus* was one of the main microbiota in both spleen-deficiency syndrome and damp-heat syndrome which might implicate it plays an important role in the outbreak of vaginitis. According to the results of a further study, it was noted that the abundance of *Prevotella* and *Pseudomonadaceae* in the BV group was greater than that in the control group, and particularly in the BV group, *Prevotella* was mainly found in damp-heat syndrome, while *Pseudomonadaceae* was mainly found in spleen-deficiency syndrome. Therefore, it could be speculated that *Prevotella* and *Pseudomonadaceae* might have a relationship with BV, and patients with BV, who have a significant abundance of both *Streptococcus and Pseudomonadaceae*, are prone to manifest spleen-deficiency syndrome, while patients who have an abundance of *Prevotella* are prone to manifest damp-heat syndrome. The analysis of the vaginal microbiota provides new ideas to effectively explain the spleen deficiency syndrome and damp-heat syndrome. Taken together, it can be concluded that *Streptococcus* is a biomarker that can differentiate between spleen-deficiency syndrome and damp-heat syndrome. Meanwhile, *Pseudomonadaceae* is a biomarker for damp-heat syndrome in BV. In addition to *Streptococcus*, *Prevotella* is also a biomarker of spleen-deficiency syndrome in BV. Hence, the present study is the first to suggest that the TCM diagnosis of spleen-deficiency syndrome and damp-heat syndrome based on the composition of the vaginal microbiota can serve as an indicator of the status of the entire body. Nevertheless, the investigators acknowledge a limitation in the present study. That is, the sample size was not sufficiently large. In future studies, a larger sample size would be employed to provide more stability.

In summary, according to the results of the NGS and bioinformatics analysis obtained from the aforementioned study on the composition of the vaginal microbiota of women with BV, and in comparison with those of healthy volunteers, it can be concluded that the composition of the vaginal microbiota can be used as a biomarker to reflect the TCM syndrome. The observations described in the present study shed light on a new direction for understanding the correlation between the composition of the vaginal microbiota and the syndrome differentiation of TCM.

## Figures and Tables

**Table 1:** Comparison of symptoms of spleen-deficiency syndrome and damp-heat syndrome [13].

	Spleen-deficiency syndrome	Damp-heat syndrome
Leucorrhea	White or pale yellow	Yellow
Tongue and coating	Tongue with tooth marks, pale tongue, white or greasy coating	Red tongue, yellow greasy
Other symptoms	Low voice and no desire to speak, lassitude, slow movement, loss of appetite, loose stool	Bit and greasy mouth, short and red urine

**Table 2:** General information of BV patients and healthy volunteers with the two different syndromes.

Characteristics	Healthy volunteers		BV patients	
	Spleen-deficiency syndrome	Damp-heat syndrome	Spleen-deficiency syndrome	Damp-heat syndrome
Number of samples	8	8	8	8
Age (mean ± SD)	28.5 ± 4.14	29.13 ± 4.39	28.88 ± 2.36	29.38 ± 2.45

## Data Availability

The data used to support the findings of this study are available from the corresponding author upon request.
